# From Guideline to Clinical Practice: Towards an Era Without Surgical Site Infections

**DOI:** 10.1002/ueg2.70151

**Published:** 2025-12-09

**Authors:** Benedikt Kaufmann, André Mihaljevic

**Affiliations:** ^1^ Department of Surgery University Hospital Tübingen Tübingen Germany

## 1 Introduction

Surgical site infections (SSI) remain one of the most common and costly complications after surgical procedures. Especially in gastrointestinal (GI) and hepatobiliary‐pancreatic surgery, the risk is high due to the bacterial colonization of the digestive tract. The risk of developing an SSI after GI surgery is around 12% worldwide. However, in contaminated surgery the risk can increase up to nearly 40% in a low‐income country worldwide [1]. The consequences are significant and include prolonged hospital stays, additional procedures, increased mortality and immense economic burdens on health systems [2]. Against this background, Sayers and colleagues updated the WHO guideline on the prevention of SSIs to establish a European version specifically for GI surgery that represents an important guideline for clinical practice [3, 4]. It updates the 2018 version and focuses on the preoperative phase, those measures that can determine the subsequent healing process before the first incision.

The updated guideline is based on a reassessment of current scientific data and formulates targeted recommendations for clinical practice following the framework of high‐quality clinical guidelines [5]. The focus is on two core recommendations:

Firstly, the use of alcohol‐based chlorhexidine solutions for skin disinfection is recommended in clean, clean‐contaminated and contaminated procedures, provided that no mucosal areas (e.g., stoma, genital area, anus) are affected. This recommendation follows the growing evidence that alcohol‐containing antiseptics show better bactericidal activity and lower reinfection rates compared to iodine‐containing solutions.

Secondly, it is recommended to stop taking corticosteroids and anti‐TNF medications before surgery. These substances, which are often used for chronic inflammatory bowel diseases, impair the immune system and have been shown to increase the risk of postoperative infections.

Previously, it was reported that a window of 4 weeks after last anti‐TNF infusion does not effect SSI [6]. For other questions, such as the comparison of different chlorhexidine concentrations or the perioperative use of biologics, no clear recommendation could be made due to a lack of sufficient data.

The economic dimension of SSIs is enormous and is estimated to cost several billion euros annually in Europe [7, 8]. In addition, SSIs are often associated with multidrug‐resistant pathogens [9, 10]. This not only leads to rising costs, but also to increasing therapeutic complexity. The guideline therefore highlights not only medical, but also health economic and social aspects. Standardized prevention measures can increase not only patient safety but also reduce the consumption of resources. This is a key point in view of the increasing cost pressure in the European healthcare system.

The importance of the updated guideline lies in particular in the consistent focus on the preoperative phase and quality assurance. Its benefits can be seen in several aspects:

– Standardization of practice: Uniform recommendations promote a Europe‐wide standardization of surgical preparation. This not only facilitates the comparability of clinical results, but also strengthens patient safety across national borders.
– Interdisciplinary cooperation: The guideline is aimed not only at surgeons, but also at all those involved in preoperative care. This establishes a unified approach to infection prevention.
– Promotion of evidence‐based decisions: By clearly stating where evidence is lacking, the guideline provides research areas where future studies should focus more specifically, such as the optimal handling of biologics or the effectiveness of new disinfectants.
– Patient orientation: Prevention means not only fewer infections but also a faster healing process and shorter hospitalization. A practice that complies with guidelines improves the quality of life of those affected directly and sustainably.

Guidelines only unfold their value through consistent implementation. It is now up to the surgical societies to put the recommendations into practice through training, quality programs, adapted preoperative checklists and prevention protocols. In addition, its implementation in national infection prevention strategies can be crucial. If hospitals see the new standards not only as an obligation but also as an opportunity, the SSI risk in GI surgery can be significantly reduced in the coming years.

## 2 Conclusion

The updated WHO guideline on the prevention of SSI in gastrointestinal surgery makes an important contribution to the prevention and reduction of SSI. Consistent implementation of the guidelines across Europe will be essential to achieving a better outcome for patients and relieve the burden on the healthcare system.

## Funding

The authors have nothing to report.

## Conflicts of Interest

The authors declare no conflicts of interest.
